# THE CLINICAL TRANSLATION OF SENSOR TECHNOLOGY TO IMPROVE PROVIDER AND PATIENT CARE: METHODS AND ACCEPTABILITY

**DOI:** 10.1093/geroni/igz038.3061

**Published:** 2019-11-08

**Authors:** Megan Huisingh-Scheetz, Qianli Xue, Jennifer A Schrack

**Affiliations:** 1 University of Chicago, Chicago, Illinois, United States; 2 Department of Medicine, School of Medicine, Johns Hopkins University, Baltimore, Maryland, United States; 3 Johns Hopkins University, Baltimore, Maryland, United States

## Abstract

Sensor-based technologies are rapidly emerging and are capable of collecting objective, dynamic, and high resolution health data not captured in the clinical setting. However, the precise clinical applications of such devices are not yet well delineated; extensive challenges to their implementation remain. The objectives of this symposium are to highlight a) opportunities for sensor technology use in clinical practice, b) implementation challenges reported by key stakeholders, and c) an NIH/VA-sponsored initiative to create an open technology research platform to improve aging technology research. Dr. Young will discuss the novel application of wearable sensors for maintaining proper posture/position during patient transfer including the generation of sensor metrics defining proper lifting technique and body mechanics. Dr. Huisingh-Scheetz will report analytic strategies for identifying frailty using wrist-worn accelerometry data collected in the free-living environment in the NIA-supported National Social Life, Health and Aging Project dataset. She will report her work relating hourly activity and between/within subject hourly activity variance to frailty. Ms. Blinka will report qualitative feedback collected from patients, caregivers, and healthcare providers about their perspectives on the utility and challenges of using sensor technology in a clinical context. Dr. Kaye will discuss ongoing developments addressing challenges to implementing technology use in clinical care, with particular attention to the Collaborative Aging Research using Technology (CART) initiative supported by the NIH and VA. Collectively, these presentations will advance sensor technology to improve healthcare delivery.

